# Role of Photoactive Phytocompounds in Photodynamic Therapy of Cancer

**DOI:** 10.3390/molecules25184102

**Published:** 2020-09-08

**Authors:** Kasipandi Muniyandi, Blassan George, Thangaraj Parimelazhagan, Heidi Abrahamse

**Affiliations:** 1 Laser Research Centre, Faculty of Health Sciences, University of Johannesburg, 17011, Doornfontein 2028, South Africa; kasim@uj.ac.za (K.M.); blassang@uj.ac.za (B.G.); 2 Bioprospecting Laboratory, Department of Botany, School of Life Sciences, Bharathiar University, Coimbatore, Tamil Nadu 641046, India; drparimel@buc.edu.in

**Keywords:** photodynamic therapy, cancer, photosensitiser, natural compounds

## Abstract

Cancer is one of the greatest life-threatening diseases conventionally treated using chemo- and radio-therapy. Photodynamic therapy (PDT) is a promising approach to eradicate different types of cancers. PDT requires the administration of photosensitisers (PSs) and photoactivation using a specific wavelength of light in the presence of molecular oxygen. This photoactivation exerts an anticancer effect via apoptosis, necrosis, and autophagy of cancer cells. Recently, various natural compounds that exhibit photosensitising potentials have been identified. Photoactive substances derived from medicinal plants have been found to be safe in comparison with synthetic compounds. Many articles have focused on PDT mechanisms and types of PSs, but limited attention has been paid to the phototoxic activities of phytocompounds. The reduced toxicity and side effects of natural compounds inspire the researchers to identify and use plant extracts or phytocompounds as a potent natural PS candidate for PDT. This review focusses on the importance of common photoactive groups (furanocoumarins, polyacetylenes, thiophenes, curcumins, alkaloids, and anthraquinones), their phototoxic effects, anticancer activity and use as a potent PS for an effective PDT outcome in the treatment of various cancers.

## 1. Introduction

Cancer is one of the deadliest diseases reported in developed as well as developing countries [1]. It is mainly characterised by the uncontrolled cell growth and development of normal cells due to genetic alterations or exposure to the carcinogenic substances. The mutation of normal cells leads to abnormal cellular proliferation and develops into tumour [2]; this can be either benign, premalignant (non-cancerous) or malignant (cancerous) [3,4]. Presently surgery, radiotherapy, and chemotherapy either as monotherapy or as combined treatments are used in the treatment of cancer. However, these treatments frequently stimulate redundant side effects [2]. Many of the current chemotherapeutic drugs are of low molecular weight with high pharmacokinetic profiles [4]. Hence, in order to achieve the bioavailability and cytotoxicity induction, the drugs are administrated in high concentrations. In photodynamic therapy (PDT), photoactive drugs are generally administered systemically, but because of the precise application of light from the laser source, the cytotoxicity is attained in the tumour location. Due to the lesser drug specificity and toxicity to healthy cells, the chemotherapeutic drugs used in cancer treatments need to be improved.

Photodynamic therapy (PDT) is a promising minimally invasive therapy for the treatment of cancer. This involves the administration of photosensitiser (PS) and subsequent excitation of PS by light irradiation at a specific wavelength. The excited PS then reacts with cellular oxygen and produces reactive oxygen species (ROS). This reaction results in oxidising the cellular macromolecules surrounding tumour cells [5]. This remedial method has been developed over the last few years [6,7], and has not only been utilised in cancer treatment, but also in dermatological [8] and ophthalmic [9] conditions, including psoriasis and age-related diseases [10,11,12]. The use of photodynamic therapy to treat cancers has gained attention around the world [13,14]. The mechanism of PDT is based on various photocatalytic reactions that induce the destruction of cancer cells, and it has been clinically used for the treatment of cancer for over a decade [5]. In the first clinical PDT study reported by Granelli et al. [15], hematoporphyrin was used as a potent photosensitiser (PS) against glioma cancer cells. PDT destroys cancer cells through three fundamentally different pathways, namely, by damaging cancer cells over time, damaging vascular tissues that supply oxygen to cells, and finally by activating host immune response systems [13,16]. Combining PDT with chemotherapy, radiotherapy, and herbal therapy could be an emerging future methodology in cancer treatment. The combination therapy has more of a tendency to reduce the side effects when compared to monotherapy regimes and can significantly lower cancer cell proliferation by improving the drug uptake [17].

Since ancient times, herbal medicine from natural products has been utilised for treating various human ailments [18]. Most current medicines are derived from various medicinal plants, and it is evident that herbal extracts and their compounds should be examined as possible active lead components in cancer drug discoveries [19,20]. Nature is a valuable reserve for medicinal plants, and many of the pharmaceutically active compounds isolated from medicinal plants have not been tested for photoactive properties. There have been few studies attempting to identify new chemical compounds with photoactivity from plant extracts that can be used as potent natural PSs [21,22,23,24,25]. Hypericin (isolated from *Hypericum perforatum*) is a recognised plant-based PS used in PDT. The in vitro and in vivo studies reported that hypericin PS activated at 594 nm could destroy cancer cell proliferation effectively. The researchers already demonstrated that the effect of herbal extracts combined with illumination could significantly reduce cancer development by prohibiting metabolic viability and proliferation cancer cells [26,27,28,29].

Due to the low or no adverse side effects, herbal products have been used for the treatment of many more ailments than synthetic drugs. Studies have shown that plant-based compounds could be used in the treatment of various cancers [30]. Many phototoxic substances were subsequently reported in various plant species that are equally efficient as of conventional PSs [31]. These studies recommend that natural compounds with photosensitising abilities can be isolated from plants and used as alternatives for conventional PSs used in PDT. In this review, the underlying principles of PDT, PSs and plant-based photoactive compounds were addressed. This review mainly focused on the anticancer activity of furanocoumarins, polyacetylenes, thiophenes, curcumins, alkaloids and anthraquinones in relation to the light-absorbing properties.

## 2. Basic Principles of Photodynamic Therapy

Photodynamic therapy involves coordination with three individual factors, namely, the photosensitiser, oxygen, and light [7]. These components are not toxic to cells individually, but when irradiated, these can initiate a photochemical reaction that generates highly reactive singlet oxygen (^1^O_2_) and cause significant toxicity, leading to cell death. PDT is normally described in two stages, the first is administration of the PS and the second stage is the irradiation. Generally, the effect of PDT is affected by the PS type, dosage, light fluence, as well as exposure time. PDT can be used either before or after chemotherapy, radiotherapy, or surgery without compromise. The clinically approved PS should not accumulate in the body and does not develop resistant cancer cells. Pain during administration and continuous photosensitisation are the major drawbacks of PDT treatment. There are three types of lights ranges from 600 to 800 nm that are commonly used in PDT, namely, blue, red, and infrared lights. Among them, blue light penetrates the tissue the least when compared to red and infrared lights. The wavelengths below 800 nm are mostly used in PDT than higher wavelengths (above 800 nm) due to their lack of photodynamic reactions. The choice of light source is commonly based on PS nature, absorption spectra of PS, location, and size and characteristics of the infected tissue [7,32].

More than 300 chemical compounds have already been identified as potential candidates to be used as PSs. Amongst these, a few were authorised for clinical application in PDT, and others were medically evaluated, whereas some are still under examination [33,34]. We have tabulated some PSs which are used in various cancer treatments in Table 1. Photosensitisers are naturally or chemically produced compound conjugated with a visible light-absorbing chromophore group with a strong chemical absorbance. Choosing the correct PS is the most important phase in PDT for a successful outcome [33,34]. The purity and the presence of a tetrapyrrole structure with good storage stability are the preferable properties of most PSs used in PDT. The potent and effective PS should have the ability to initiate a photodynamic reaction after irradiation with 600–800 nm lights and should not cause any toxicity under dark conditions. It should be easily distinguishable from the body with no or minimum phototoxic side effects [35]. The better diffusion of PS through the cells after long administration might contribute to the effectiveness of PDT [36]. The production of a significant amount of ROS after irradiation that induces apoptosis with less inflammation is most likely to be a suitable PS for PDT application [37,38]. 

**Figure 1:**
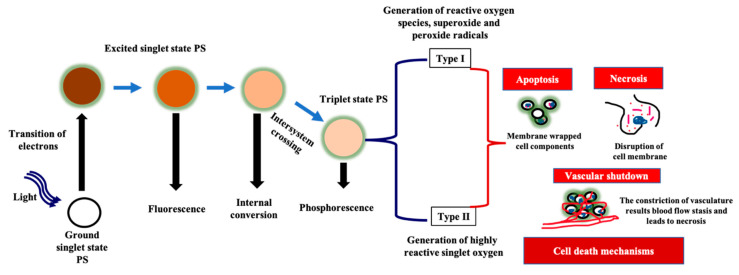
The general mechanism of photodynamic therapy.

When a PS is subjected to a particular wavelength light, the electron of the outermost orbital will be shifted from the ground state (S0) to the first excited state (S1). Subsequently, the electromagnetic propulsion switches the molecule to an excited triplet state (T1) with a longer life span (Figure 1). In each of these excited states, PSs are quite unstable and lose their energy in the form of fluorescence, phosphorescence, and internal heat conversion. PSs in the T1 state may react photochemically in any of the two pathways. In the type 1 pathway, the excited PS reacts through an electron transfer process with the surrounding oxygen, which ultimately leads to generation of reactive oxygen species (ROS). Such free radicals communicate readily with the biomolecules (lipids, peptides, proteins, and nucleic acids) and destroy them [39,40]. In contrast, in the type 2 pathway, the energy is directly transferred from the T1 state of the PS to the S0-state oxygen. This results in the ground-state PS transformation and excited-state singlet reactive oxygen. The disruption caused by PDT is local because both singlet oxygen as well as free radicals have a short half-life between 10–300 nanoseconds and a small diffusion distance of 10–55 nm [41].

**Table 1:** List of photosensitisers used in photodynamic therapy of various cancers.

Photosensitiser	Commercial Name	λ max (nm)	Structure	Type of Cancer	Reference
**First-Generation Photosensitiser**					
Hematoporphyrin derivatives	Photofrin Photoheme	630	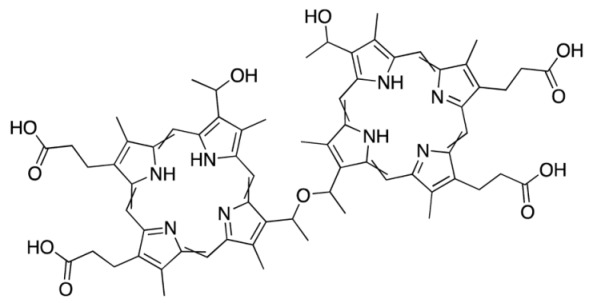	Lung, bladder, skin, cervical, breast cancer.	[17,42]
**Second-Generation Photosensitisers**					
5-Aminolevulinic acid	Levulan Alasens	635	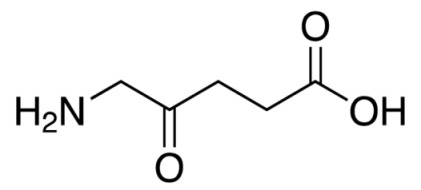	Bladder, skin, lung, ovary and gastrointestinal cancer.	[43,44,45]
Meta-tetra(hydroxyphenyl) chlorin	Foscan	652	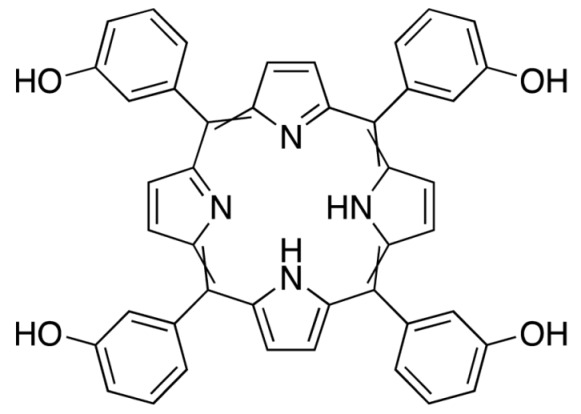	Approved drug for the treatment of bronchial and oesophageal cancers.	[46,47,48]
Chlorin e6	MACEDACEPhotoditazine	664	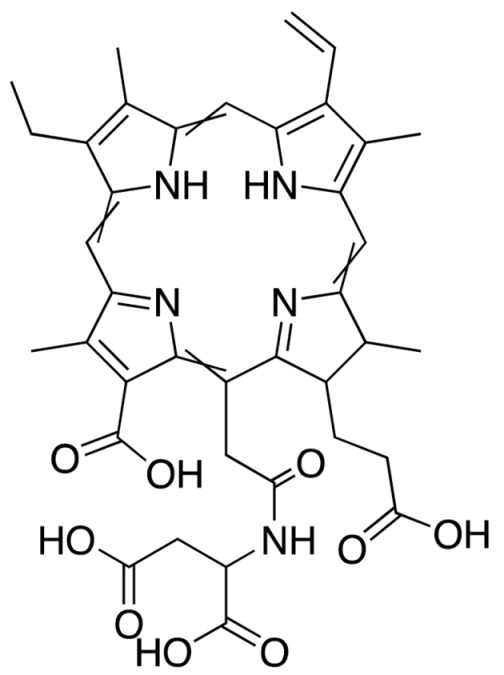	Gynaecological diseases, prostate cancer, fibrosarcoma, Liver, brain, lung, and oral cancers.	[49,50,51]
Benzoporphyrin	Visudyne	690	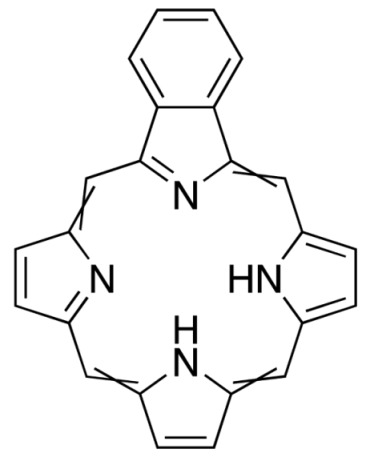	Prostate and skin cancer.	[52,53]
Texaphyrins	Lutrin, Antrin, Optrin, Xcytrin	720–760	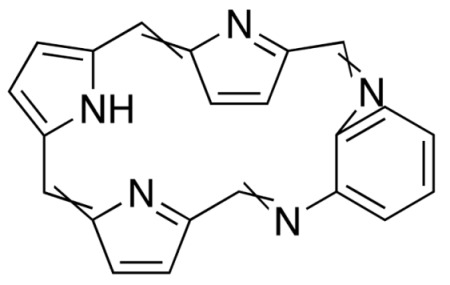	Hepatocellular cancer, leukaemia, nasopharyngeal carcinoma, colon, prostate, bronchial and oesophageal cancers.	[54,55,56,57,58]
Phthalocyanines	Photosense	640–690	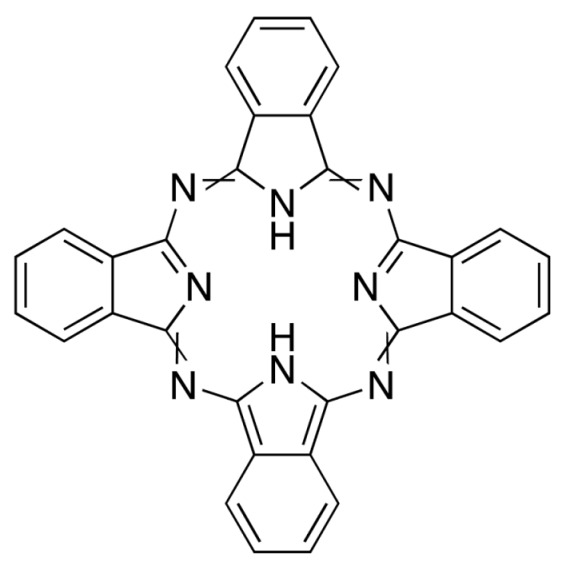	Breast, cervical, skin, lung, liver, colon and gastrointestinal cancers.	[17,59,60,61]
Purpurins	Purlytin	660	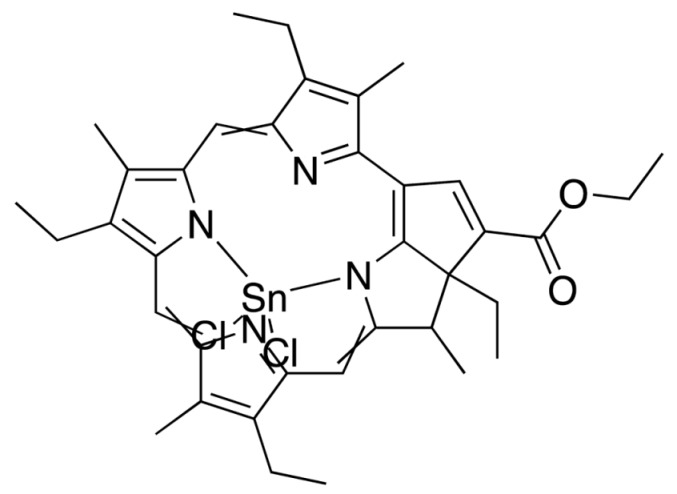	Breast cancer, prostate cancer and Kaposi’s sarcoma.	[62,63,64]

## 3. PDT’s Cancer Cell Death Mechanism

PDT’s cancer cell death mechanism starts after the activation of administrated PS by a specific wavelength of light. The PS’s hydrophilic, hydrophobic, and ionic charge-related interaction nature plays an important role in the targeting of particular cancer cell receptor (globulins and Low-Density Lipoprotein (LDL) receptors) [34]. After the activation of PSs, the cancer cell death mechanism might occur in three main pathways (Figure 1), namely, apoptosis, necrosis, and autophagy [33,65,66]. However, the level of cell death induced by PDT may be affected by various aspects, including subcellular localisation, bioavailability, the physicochemical nature of the PS, the cellular oxygen concentration, as well as the applied light intensity and wavelength [67]. In general, the light-absorbed PS interacts with cellular oxygen and highly produce ROS (hydroperoxides, superoxide, or hydroxyl radicals) as well as singlet oxygen (^1^O_2_). These produced ROS can induce cancerous cell death via the above-mentioned mechanisms. Both type 1 and 2 reactions may occur separately or in combination, but type 1 (generation of ROS followed by the apoptotic cell death mechanism) is commonly exhibited by most approved PSs [67].

## 4. PS from Natural Resources

The effectiveness of PDT is mainly based on the PS; it should possess all the properties of the PS as previously explained. The PS can be divided into first- and second-generation types. Hematoporphyrin and its derivative Photofrin^®®^ were classified as first-generation PSs. After extensive studies, new and improved second-generation PSs, such as Levulan^®®^, Alasens^®®^, and Foscan have been introduced for PDT application (Table 1). Although these are widely used for various cancer treatments, their clinical usage is limited by various drawbacks such as lack of chemical purity, a longer half-life, accumulation in tissues and poor ability in relation to depth of tissue penetration [31,32,33,34,35,36,37,38,39].

**Figure 2:**
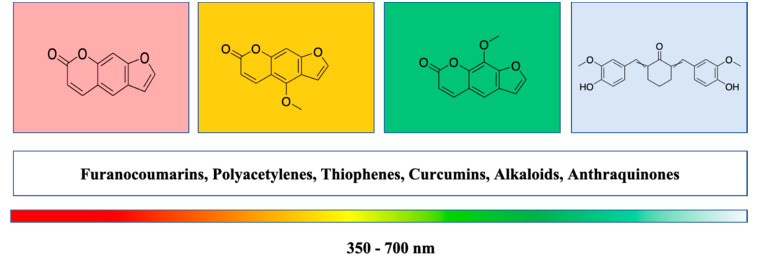
Phototherapeutic window of natural compounds.

Subsequently, there are some research reports on PSs with potent pharmaceutical properties to overcome the shortcomings of first- (Porphyrin based sensitisers) and second-generation (non-porphyrin derivatives) PSs [35,36,37]. These drawbacks of current PSs specifically imply the need for new PSs as anticancer agents from natural resources. The discovery of new PS compounds with anticipated pharmacological properties and clinical application is an inspiring task. Recently, a greater number of plant-based compounds have been reported for their anticancer activity, and these compounds are pharmaceutically very important for the development of potent drugs. The use of light to activate the bioactivities of natural products is generally called photopharmacology (a combination of photophysics and photochemistry). The absorption of lights (λ < 350 nm) by a molecule mainly depends on the chromophore compound attached (Figure 2) [36,37]. This review presents an overview of natural photoactive compounds as potent third-generation photosensitisers in the improvement of PSs in relation to their prospective application in cancer treatments.

## 5. Natural Photoactive Compounds from Plants

The search for the natural compounds as efficient PSs has been progressively moving forward because of the side effects caused by current synthetic drugs. The advanced isolation, identification and characterisation techniques improved the extraction of desirable compounds from plants. Recently, using these advanced techniques, the isolation of natural photoactive compounds has become easy. Although there have been few studies attempting to identify new chemical compounds with photoactivity from plant extracts, this review discusses the photoactivity as well as the anticancer activity of some plant-based compounds such as furanocoumarins, polyacetylenes, thiophenes, curcumins, alkaloids and anthraquinones (Table 2).

### 5.1. Furanocoumarins

**Figure 3:**
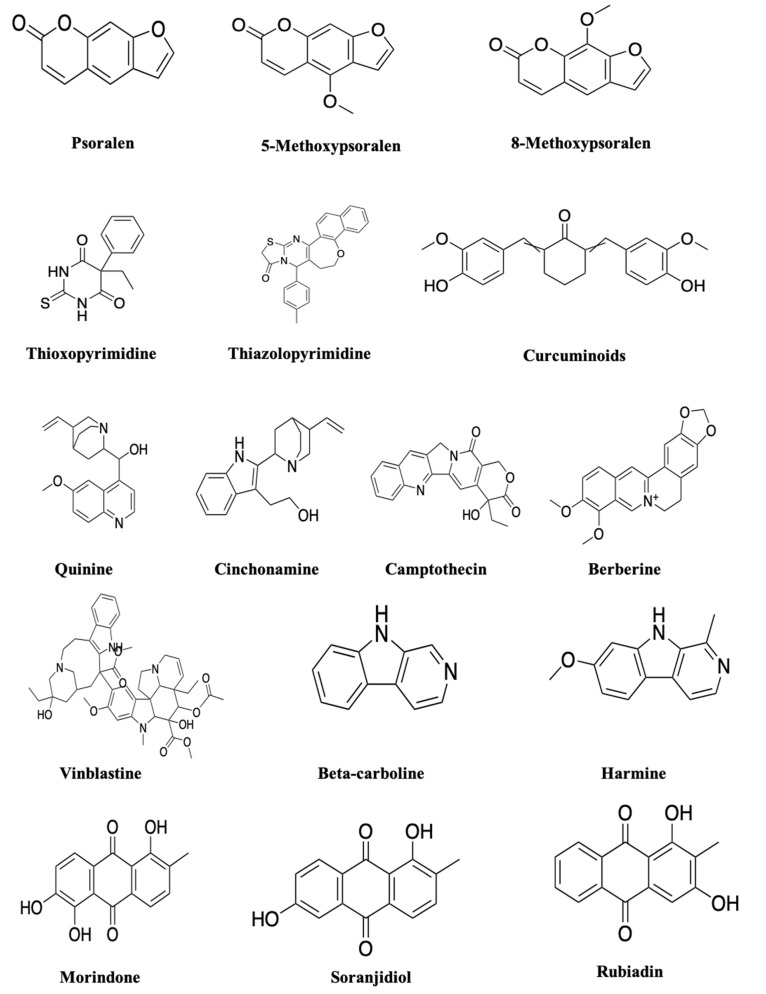
Natural photoactive compounds presented in this review.

**Figure 4:**
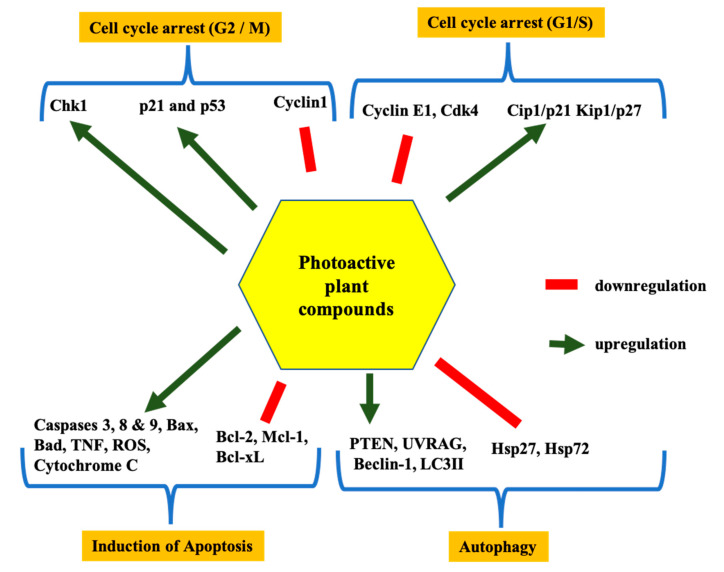
Common molecular targets of major photoactive compounds.

The secondary metabolites, furanocoumarins (FC; Figure 3), are mostly present in higher plants. The photoactive furanocoumarins were mainly composed of a linear core, and the biological distribution, photochemistry and phototoxicity mechanisms of FC after PUVA (psoralen and long-wave ultraviolet radiation) irradiation were reported in previous study [92]. In terms of phototherapy, psoralen is activated in the wavelength range of 300–400 nm ultraviolet radiation to treat psoriasis, dermatitis, eczema, and other skin problems [92]. Over a few years, many researchers reported anticancer activity of FCs against various types of cancer such as breast, skin, and leukaemia. FCs modulate several pathways inducing cancer cell death by inhibiting signal transducer and activator of transcription 3 (STAT3), nuclear factor-κB (NF-κB), phosphatidylinositol-3-kinase and AKT protein expression (Figure 4). These pathways play a key role in tumour development through regular activation of several inflammatory genes. Studies show that FC displayed potent activity against breast cancer development by inhibiting STAT3 protein expression [93]. Panno et al. [92,93], demonstrated inhibition of breast cancer cell growth in a dose-dependent manner through activation of p53 and Bax, leading to the cleavage of caspase 9. In contrast, in leukaemia cells, FC inactivated the JAK (Janus-activated kinase), protein c-Src, and STAT3, and downregulated Bcl-xl and Bcl-2 proteins which are responsible for apoptosis [93,94,95,96]. The enhanced activity against the malignant melanoma cell line (A375) after UV irradiation of plant extracts containing FC also supported the possible photoactive nature of FCs [93,94,95,96]. The linear forms of furanocoumarins like psoralen and its derivatives 5-methoxypsoralen (5-MOP) and 8-methoxypsoralen (8-MOP) are reported to increase the cytotoxicity after irradiation by ultraviolet light in the 320–400 nm wavelength range against cutaneous T-cell lymphoma [97,98], and photoactivated psoralens induce apoptosis by forming adducts with DNA. This leads to the activation of p21waf/Cip and p53 and subsequently leads to cell death by the release of mitochondrial cytochrome c. The photoactivation of psoralen can also cause cell death by blocking oncogenic receptor tyrosine kinase signalling and the PI3K pathway by interfering with efficient recruitment of effector Akt kinase to the activated plasma membrane [92,93,94,95,96,97,98,99,100,101,102,103,104]. PUVA treatment was found effectively against B16F10 murine melanoma cells by cell cycle arrest in G2/M phases [93,94,95,96].

### 5.2. Polyacetylene and Thiophenes

These group compounds are characterised by a triple bond carbon–carbon molecule [75] and thiophenes compounds. Generally, the aliphatic compounds conjugated with three or more acetylenic bonds are considered phototoxic in nature. Among these, polyacetylenes compounds can produce ^1^O_2_ under irradiation and thiophenes can provide high photo yield, leading to type 2 PDT reaction yields [105]. The polyacetylene and thiophenes compounds were reported to be activated or excited at a wavelength range of 314–350 nm absorbance maximum for the relevant photobiological effects [75]. The derivatives of these compounds were reported with a variety of potent biological activities, including analgesic, anti-inflammatory, antitumour, and antimicrobial activities. Some of the derivatives substituted by pyrimidines show antimicrobial, anti-inflammatory, and antitumour activities. A few numbers of thiophenes were reported for their cytotoxic effects against human cancer cell lines. *Echinops grijisii* root-derived thiophenes exhibited cytotoxicity against HL-60, K562 and MCF-7 cells [106]. Notably, derivatives such as thioxopyrimidine and thiazolopyrimidine were reported to possess anticancer activities against MCF-7 (breast adenocarcinoma), NCI-H460 (non-small cell lung cancer), and SF-268 (CNS cancer) cells. These acetylenic compounds and derivatives when combined with PDT might improve the efficacy of various cancer treatments [107,108,109,110,111,112]. The UV irradiation of some thiophenes also showed increased cytotoxic activities [113]; this might be due to their instable nature under UV radiation. Hence, the UV irradiation of polyacetylene and thiophene compounds can form free radicals that would induce cell death. Due to the ability to produce ROS after irradiation, these compounds can be used as an alternative PS from natural sources.

### 5.3. Curcumins 

Curcumin (CU) is a plant-based therapeutic compound isolated from rhizome of *Curcuma longa* of the Zingiberaceae family. Curcuminoid is one of the most extensively studied plant-derived bioactive compounds [114]. Since the 1980s, the photobiological potential of CU was of great interest [115,116], and studies described CU as a desirable, highly promising photosensitiser [115,117,118,119,120,121]. The foremost property of CU is that it is biologically safe even at higher doses, and it can be easily produced on a large scale [117,122]. The photobleaching analysis reported the degradation profile of curcumin derivatives and its ability to produce singlet oxygen species [123]. CU was characterised by an absorption spectrum of 300–500 nm with a high extinction coefficient. This suggests that CU can induce a strong phototoxic reaction even at lower concentrations [124,125,126]. Curcumin is considered as a potential anticancer agent and inhibits cancer cell proliferation in breast, lung, colon, kidney, ovary, and liver cancers [114]. The in vitro and in vivo anticancer activity of curcumin has been proved by inhibition of various transcription factors such as NF-κB, AP-1, VEGF, iNOS, COX-2, 5-LOX, MMP-2, MMP-9 and IL-8, which are mainly responsible for angiogenesis and tumour growth [127,128]. The administration of curcumin significantly reduced the expression of the CDK4/cylin D1 complex by inhibiting p53 expression and causing the apoptotic process by inducing ROS generation. Furthermore, the enhanced antitumour activity was noted after UVB irradiation of curcumin by caspase activation on HaCaT (human keratinocyte cell) cells. It was also efficient against MCF-7 breast cancer cells at 30 J/cm^2^ [77,78]. These data suggested that curcumin may act as a potent anticancer agent by preventing cancer progression, migration and invasion [129,130,131]. Dovigo et al. [132] found the light absorption ability of CU in the range of 300 and 500 nm with a maximum absorption at 430 nm, which might support its usage as a PS. The ROS-inducing and anticancer ability of CU makes it a potent candidate as a natural PS [133]. Nevertheless, Chan and Wu [134] observed that the photoactive nature of CU on human epidermal A431 carcinoma cells and the higher amount of CU also affect the irradiation penetration [132,134,135]. The irradiation of CU under a 290–320 nm UVB light source with the fluence of 100 mJ/cm^2^ induced apoptosis in HaCaT keratinocyte cells [78]. Based on the above reports, CU can be used as a natural PS, and it can achieve high efficacy at a low concentration when combined with PDT. The existing PDT and photoactive reports on CU suggest that CU can be used as a potential and promising natural PS in PDT. In conclusion, CU can be a potent photosensitiser in the treatment of cancer and skin infections. Therefore, investigating the photodynamic potential of CU derivatives in terms of higher absorption and extinction coefficient will contribute to the increased efficacy of photodynamic toxicity.

### 5.4. Alkaloids

Alkaloids, a diverse secondary metabolites group from higher plants, contain a heterocyclic structure with a nitrogen atom in the ring [136]. Nitrogen-containing alkaloids are normally photoactive in nature, e.g., quinine and cinchonamine. The alkaloids were reported for many significant properties, such as analgesic and anticancer activity [136,137,138,139]. The alkaloids camptothecin and vinblastine are few alkaloids were successfully utilised as chemotherapeutic drugs [138,140]. The anticancer activity of alkaloids was proved by different studies by means of disturbing tumour progression by induction of cell cycle arrest at the G1 or G2/M phases, regulating cyclin-dependent kinase (CDK) and promoting apoptosis as well as autophagy in tumour cells. Furthermore, these compounds induce apoptosis by regulating Bax, Bcl-2, Bcl-xL, NF-κB and various caspase proteins [140,141,142,143]. In addition, the combination of alkaloids with chemotherapeutic drugs and irradiation also enhanced the biological activities [144,145]. Furthermore, alkaloids induce the formation of intracellular ROS in cancer cells, which leads to the destruction of cancer cell metabolism [140,141,142,143]. The photochemically best-known alkaloid is berberine; Luiza Andreazza et al. [146], Bhattacharyya et al. [147] and Inbaraj et al. [81] reported the antitumour activity of berberine upon UV and blue light irradiation. The irradiation of berberine at 410 nm proved to be effective in controlling brain cancer cell growth [146]. Beta-carboline and harmine are also a noticeable alkaloid with a photoactive nature and are reported to produce a significant amount of ROS after irradiation [148], which is considered an important feature of potent PSs. The photoactivity of harmine was proved by the UVA (long-wave ultraviolet radiation) irradiation against tumour cell lines [148]. Berberine was extensively investigated as a potential photosensitising agent for PDT [149,150,151]. The fluorescent active nature of berberine is indicated for its efficiency in PDT [149]; thus, berberine and its associated alkaloids can be used as a new candidate for photodynamic therapy [150]. Different studies have proved the photosensitising as well as ROS generation ability of alkaloids in the presence of a light source [151]. Therefore, berberine can be studied as a natural photosensitiser in PDT applications with minimal side effects.

### 5.5. Anthraquinones (AQ)

Anthraquinone are the largest group among natural quinones from higher plants, which, including naphthoquinones and benzoquinones, includes over 700 compounds, including emodin, physcion, catenarin and rhein [152,153]. The hydroxylation pattern, however, dictates the possibility of AQs’ photopharmacological properties. Notably, AQs’ aminoanthraquinone derivatives were studied extensively for their photoactive properties among the plant compounds due to their UV/vis absorption and photosensitising nature [154,155]. The AQs were reported as kinase and tyrosinase inhibitors as well as cytotoxicity agents. The *M. elliptica* AQs such as morindone, soranjidiol and rubiadin were also reported for their antitumour activity against lymphocytic leukaemia (P-388) cells [156]. The anthraquinones isolated from *H. pustulata* leaves and stem exhibited photosensitising properties by generation of singlet oxygen and/or superoxide anion radicals [157]. Comini et al. [158] reported that irradiation of AQs (soranjidiol and rubiadin) under visible radiation of 380–480 nm can promote the anti-proliferative effect on MCF-7 breast cancer cells. In addition, Montoya et al. [157] and Vittar et al. [159] also reported photosensitisation effects of AQs in Balb/c mice and their leukocyte-inhibiting ability in a dose-dependent manner by inducing apoptosis, necrosis, or autophagy. These study results show the photoactive nature of AQs to inhibit the proliferation of cancerous cells. Based on the previous studies and the above data, molecular targets responsible for the anticancer activity of AQs and major phytocompounds are summarised in Figure 4.

## 6. Theorical Studies for Assessing the Photoactivity of Natural Compounds

The development of various antitumor compounds with different molecular targets initiated an exciting field of investigation with recently developed theoretical studies. The theoretical studies including density functional theory (DFT) and time-dependent density functional theory (TD-DFT) were used to assess a series of photophysical properties, including absorption spectra, excitation energies (singlet and triplet) and spin–orbit matrix elements. All the reported compounds are potential UVA chemotherapeutic agents which require the lowest triplet-state energy for producing highly cytotoxic ROS [160,161].

## 7. Advantages and Scope of Natural PSs

The anticancer property of many plant extracts and bioactive compounds have been analysed, but not so much in terms of as sources of photosensitisers. Selecting proper PSs is the first step in PDT, and, to date, only a few PSs are clinically approved, such as Photofrin, Foscan and Levulan. The present study explored the common photoactive nature of various phytocompounds. Many of the natural photoactive compounds were reported for their non-toxicity against normal cells and toxicity towards cancer cells. The important property of a PS is the nontoxic nature during the absence of light. The increasing activity of extracts or phytocompounds after irradiation by light makes them good photosensitising candidates for PDT. Another important feature that makes photoactive plant compounds suitable photosensitisers is their absorption maxima at 400–700 nm, which is biologically compatible. The selective nature of these compounds is important in clinical PDT to overcome side effects. Future studies are warranted to isolate and evaluate these specific photoactive compounds from plants to be used as a potent PSs for PDT for cancer and related disorders [162,163].

## 8. Conclusions and Future Perspectives

As discussed in this review, plant-based photoactive compounds can be used as a natural PSs in PDT application. There are wide range of unknown natural compounds with different photoactive and phototoxic properties. This review summarises and encourages researchers to identify and elucidate natural photoactive plant-based compounds and to use them as alternatives for the synthesis PSs for a better PDT outcome. Furthermore, discovering natural phototoxic agents as PSs will be helpful to reduce toxicity and side effects and improve selectivity. In conclusion, use the plant-based PSs in PDT typically causes less and minimal adverse effects than other treatments that are commonly used in cancer therapies.

## Figures and Tables

**Table 2:** List of plant-based natural photoactive compounds with known photoactivity.

Name	Absorption Maxima	Chemical Property and Groups	Natural Sources	Possible Mode of Action	Reference
Furanocoumarins	333 nm	Aromatic compounds possessing a furan ring.	*Angelicae dahuricae, Tetradium daniellii, Glehnia littoralis, Heracleum persicum, Syzygium Sps, Ruta graveolens, Ficus sps.*	DNA intercalation under dark type 2 PDT reaction. Crosslinking and adduct formation with DNA and RNA. Cell membrane damage.	[68,69,70]
Polyacetylenes and Thiophenes	488 nm	Furanoacetylenes thiarubrines, thiophenes, polyacetylene (aliphatic compounds with more than three conjugated triple bonds), thiophenes (aromatic acetylenes; e.g., phenylheptatriyne).	*Asteraceae* spp, *Heliopsisa*, *Rudbeckia* spp, *Arnica, Centaurea scabiosa, Tagetes erecta*, *Porophyllum obscurum, Echinops*, *Bidens, Ambrosia chamissonis, T. minuta, E. latifolius*, *E. sgrijissi,* *Rhaponticum uniflorum.*	Membrane damage or erythrocyte leakage; type 1 and type 2 PDT reaction, as well as type 1 and 2 PDT mixed reaction.	[71,72,73,74,75]
Curcumins	420–480 nm	Dicinnamoylmethane, curcumin, curcuminoids, demethoxycurcumin, bisdemethoxycurcumin.	*Curcuma longa.*	Cell membrane is the primary target of curcuminoids. Induction of caspase-mediated cell death.	[76,77,78]
Alkaloids	360 nm	Chinolin alkaloids, pterins, benzylisoquinolines, beta-carbolines, harmine.	*Guatteria blepharophylla*, *Berberis vulgaris, Sanguinaria Canadensis*, *Mahonia aquifolium* *Peganum harmala*, *Indigofera tinctoria.*	Photo-oxidises histidine and tryptophan, resulting in DNA crosslinking. Photooxidation, type 1 PDT mechanism and targets mitochondria.	[79,80,81,82,83,84,85,86,87]
Anthraquinones	437 nm	Hydroxyanthraquinones, rhein, physcion, emodin, rubiadin, damnacanthol, soranjidiol, alizarin, purpurin, rubiadin, aloe-emodin, 1,5-dihydroxy przewalsquinone B, ziganein, uredinorubellins, caeruleoramularin, hypericin, cercosporin, elsinochromes A-C pleichrome, hypocrellin.	*Polygonum cuspidatum, Heterophyllaea pustulata, H. lycioides* *Aloe vera, Rheum palmatum*, *Rumex crispus* *Polyathia suberosa, Dactylopius coccus, Xanthoria parietina, Drechslera avenae,* *Ramularia collo-cygni.* *H. perforatum*, *Fagopyrum esculentum.*	Type 1 and 2 PDT action.	[88,89,90,91]
